# Effects of a Soft Robotic Exoskeleton for Gait Training on Clinical Outcomes in Patients With Parkinson Disease: Randomized Controlled Pilot Study

**DOI:** 10.2196/82629

**Published:** 2026-04-28

**Authors:** Shiying Bu, Xiaoqing Luo, Shuangfang Li, Yuqin Shi, Lingjing Jin, Qing Zhao

**Affiliations:** 1 Shanghai YangZhi Rehabilitation Hospital (Shanghai Sunshine Rehabilitation Center), Tongji University School of Medicine, 2209 Guang Xing Road, Songjiang District, Shanghai, 201619, China, 86 13817857620

**Keywords:** soft exoskeleton robot, walking training, Parkinson disease, balance function, cognitive function, activities of daily living

## Abstract

**Background:**

Balance and gait disorders in Parkinson disease (PD) impair motor function and quality of life.

**Objective:**

Evidence on soft exoskeleton robots (SERs) for PD rehabilitation is limited. This study evaluated the impact of SERs on motor dysfunction in PD.

**Methods:**

A total of 56 people with PD (July 2023 to May 2024) were randomized to 2 groups: the control group (n=25, 44.6%) received conventional rehabilitation, and the experimental group (n=31, 55.4%) received conventional rehabilitation combined with SER training (ChiCTR2500111990). Training occurred 5 times per week for 20 minutes each session over 4 weeks. Primary outcomes included gait speed and stride length, while secondary outcomes assessed the percentage of swing phase, ankle joint range of motion, Unified Parkinson Disease Rating Scale total and motor scores, and Montreal Cognitive Assessment. Paired sample *t* tests (2-tailed) were used for within-group pre- and postintervention comparisons, and independent sample *t* tests (2-tailed) were used for between-group comparisons. Correlation analyses were conducted between gait parameters and improvements in ankle mobility.

**Results:**

After 4 weeks, the experimental group showed significant improvements in gait and balance. Specifically, left stride length increased by a mean of 0.15 (SD 0.16; 95% CI 0.09‐0.21) m (*P*<.001), right stride length by a mean of 0.15 (SD 0.15; 95% CI 0.10‐0.21) m (*P*<.001), left ankle dorsiflexion by a mean of 2.84 (SD 1.46; 95% CI 2.32‐3.36) degrees (*P*<.001), left swing phase percentage by a mean of 1.56% (SD 3.05%; 95% CI 0.44‐2.68; *P*=.01), and right swing phase percentage by a mean of 1.6% (SD 2.72%; 95% CI 0.62‐2.62; *P=*.002). The Unified Parkinson Disease Rating Scale Part III total score decreased by a mean of 2.80 (SD 3.98) points, and balance subscale scores decreased by a mean of 0.40 (0.58) points (*P*<.001). Montreal Cognitive Assessment scores increased by a mean of 1.23 (1.23; 95% CI 0.77‐1.68) points (*P*<.01), and Barthel Index scores increased by a mean of 6.84 (7.14; 95% CI 4.22‐9.46) points (*P*<.001). Other measures such as balance reaction time, reaction speed, maximum movement distance, and movement direction control showed significant improvement (*P*<.01). Compared to the control group, the experimental group demonstrated greater improvements in gait speed (*P*=.04), balance reaction time (*P*=.04), and maximum movement distance (*P*=.048). Correlation analysis revealed that improvements in left ankle dorsiflexion were positively correlated with improvements in gait speed, stride length, and swing phase duration (*P*<.05).

**Conclusions:**

SER-assisted training significantly improves gait, balance, and PD symptoms. Our work integrates multidimensional assessments (gait analysis, balance metrics, and clinical scales) and reveals that gains in ankle mobility directly correlate with gait improvements, suggesting a key mechanism. This study contributes by establishing SER as an effective adjunct to conventional therapy, supported by comprehensive quantitative data.

## Introduction

Parkinson disease (PD) is the second most common neurodegenerative disorder, with major motor symptoms including bradykinesia, resting tremor, rigidity, and postural-gait disturbances, which significantly affect mobility and quality of life [1,2]. Gait dysfunction is a prevalent manifestation of PD, with more than 70% of patients showing abnormalities such as reduced step length, freezing of gait, and diminished arm swing within 5 years of diagnosis. The incidence of freezing of gait increases from 37.9% in early-stage PD (disease duration ≤5 y) to 64.6% in late-stage PD (disease duration ≥9 y), reflecting the progressive decline in gait function [3]. Treatment outcomes for PD-related gait disorders remain unsatisfactory, with substantial variability in the effectiveness of pharmacological treatments and surgical interventions, such as deep brain stimulation [4-6]. Patients also frequently experience postural instability, which is marked by abnormal balance reflexes, reduced stability limits, and disrupted compensatory strategies [7]. A study comparing balance function in healthy individuals and people with PD (H&Y stages 1‐3) using clinical tests and postural recording found that people with PD had significantly reduced maximum movement distance and slower movement speed during the stability limit test compared to healthy controls (*P*<.05) [8]. Additionally, the stability limits during static standing were significantly smaller in people with PD than in the healthy controls (*P*<.05) [8]. These objective abnormalities confirmed a significant impairment in postural control in this population. A multicenter cohort study revealed that 34% of people with PD developed detectable balance deficits within 2 years of diagnosis, which increased to 71% at the 10-year follow-up and reached 92% after 15 years [9]. Biomechanical analysis showed reduced ankle joint torque and decreased trunk counterrotation during balance recovery tasks, indicating impaired use of ankle strategies compared to healthy individuals [10].

The pathological mechanisms underlying gait disturbances in PD stem from dopaminergic depletion within the basal ganglia-thalamocortical circuit, resulting in impaired automatic motor control [11]. Under normal physiological conditions, rhythmic gait initiation relies on the coordination of the nigrostriatal pathway to synchronize limb movement and facilitate center-of-mass transfer [12]. In people with PD, the reduced activity of the direct pathway and excessive activation of the indirect pathway lead to enhanced GABAergic inhibition of the pedunculopontine nucleus, clinically manifesting in characteristic Parkinsonian gait abnormalities such as shortened stride length, loss of reciprocal arm swing, and motor freezing [13]. To compensate, patients adopt a high-frequency shuffling gait strategy, which inadvertently exacerbates energy expenditure and postural instability. The pathological mechanisms of postural control deficits involve degeneration of the basal ganglia-brainstem-cerebellar pathway, leading to suppressed vestibulospinal reflexes. This results in axial rigidity and reduced joint range of motion (muscle stiffness and bradykinesia), while damage to the dopaminergic system impairs sensorimotor integration, particularly when visual and vestibular information conflict. In such cases, patients exhibit increased postural sway and reduced stability limits [14-16]. These mechanisms collectively contribute to the progressive worsening of gait parameters in people with PD.

In recent years, soft robotic exoskeletons (SREs) have rapidly entered clinical applications due to their lightweight, real-time performance, and ease of use. By using biomimetic lightweight drive designs, SREs maintain joint mobility while reducing metabolic costs, overcoming the limitations of rigid exoskeletons [6]. Representative flexible exoskeleton robots include the Myosuit from the United States, which was tested by a Swiss team in a study where 12 participants with various movement disorders, each capable of walking at least 10 meters, underwent 5 sessions of 45-minute Myosuit training. Five participants showed a significant increase in walking speed postintervention compared to baseline levels [17]. Another example is Japan’s wearable Stride Management Assist exoskeleton robot, which was studied in a trial involving 8 individuals with PD at H-Y stages 1‐2.5. The study involved robot-assisted walking training conducted for 4 weeks, 45 minutes per session, 3 times a week. The results showed improvements in walking speed, stride length, and hip joint mobility, along with a decrease in gait time and improvements in long-range autocorrelation measures associated with PD gait variability. Additionally, hip joint maximum peak torque and energy consumption decreased, while confidence in balance during activity improved [18]. In China, the Yrobot RelinkTM muscle exoskeleton robot was evaluated in a study by Xie et al, which demonstrated that it was superior to conventional training in improving walking speed and recovery in the 10-m walk test and 6-minute walk test for patients with subacute stroke. All these exoskeletons simulate the working principles of human muscles to assist joints and provide adaptive power assistance to the body [19]. Preliminary evidence suggests that SRE-assisted training can improve gait parameters in patients with neurological diseases. However, a meta-analysis of rigid exoskeleton interventions for PD (14 RCTs, n=572) shows significant improvements in balance function (Berg Balance Scale) [20], although the efficacy of SREs specifically designed for PD gait remains unclear.

It is noteworthy that existing studies on exoskeleton-assisted gait training in PD have demonstrated significant heterogeneity in therapeutic effects, which may be related to differences in device types and intervention protocols. This pilot randomized controlled trial (RCT) aimed to investigate the clinical efficacy of an ankle-targeted SRE system in improving gait abnormalities and balance impairments in people with PD.

## Methods

### Study Setting

This study is an RCT conducted from July 2023 to May 2025 at Shanghai Yangzhi Rehabilitation Hospital in China, which screened and recruited 56 patients with PD.

### Participants

The inclusion criteria were (1) idiopathic PD, (2) Hoehn-Yahr stage 2‐3, (3) evident gait abnormalities, (4) age between 50 and 80 years, and (5) stable medication, with no changes expected in the next 3 months. The exclusion criteria were (1) severe psychiatric disorders; (2) other terminal illnesses, including but not restricted to cancer; (3) history of substance abuse or alcoholism; (4) inability to understand or cooperate with the study; (5) pregnant or lactating women; and (6) participation in other clinical trials. All participants were fully informed of the study content and potential risks and voluntarily signed the informed consent form to confirm their participation.

### Ethical Considerations

This study was approved by the Ethics Review Committee of Shanghai Yangzhi Rehabilitation Hospital. The study protocol was approved by the Yangzhi Institutional Review Board (IRB). The authors have obtained a letter from the IRB confirming that the protocol and design of the submitted manuscript are identical to the version that received approval before the trial started. This confirmation has been mentioned here and provided to the editorial office for review.

This study was reviewed and approved by the relevant IRB to ensure that all procedures involving human subjects adhered to ethical guidelines. Informed consent was obtained from all participants for the primary data collection, and for secondary analyses, the original informed consent explicitly permits such use without requiring additional consent. All participant data were anonymized and deidentified to safeguard privacy and confidentiality. In cases where identifiable data were used, appropriate measures such as data encryption and restricted access were implemented to ensure security. No compensation was provided to participants for their involvement. Furthermore, all images or supplementary materials included in the manuscript do not identify any individuals. The photograph included in this manuscript shows 2 individuals, but no identifying features (eg, face, name, or other personal information) are visible, and they cannot be identified from the image.

### Trial Registration

This trial was retrospectively registered with the Chinese Clinical Trial Registry (ChiCTR) [21] under registration number ChiCTR2500111990.

### Reporting Guidelines

This RCT is reported in accordance with the CONSORT (Consolidated Standards of Reporting Trials) 2025 statement (Checklist 1) [22] and eHealth guidelines [23].

### Sample Size

The sample size calculation was based on data from previous trials, with walking speed as the primary efficacy end point. A 2-tailed significance level of 0.05 and a power of 0.9 were used [24]. The sample size was calculated using the PASS15 sample size formula [25,26], resulting in a required sample size of 26 participants for the experimental group and 26participants for the control group. Assuming a 20% dropout rate, the final sample size for each group was adjusted to 32 participants.

### Randomization

#### Sequence Generation

After the baseline assessment, people with PD were randomly assigned to the SRE group or conventional rehabilitation (CR) group in a 1:1 ratio using a computer-generated random number table.

#### Allocation Concealment Mechanism

The allocation sequence was stored in a sealed envelope by an investigator who was not involved in training or assessment.

#### Implementation

Upon the registration of a new eligible participant, an envelope was randomly selected, and the assigned group was communicated to the therapist.

### Blinding

Both pre- and postintervention assessments were conducted by the same certified therapist, who was blinded to the group allocation. To ensure interrater reliability, a second certified therapist also conducted parallel assessments, and the scores from both therapists were compared. This process was implemented to maintain consistency and minimize bias in the subjective outcome measures.

### Interventions

This study used the Yrobot Relink flexible exoskeleton robot (Yrobot Inc) [27] to assist in gait rehabilitation for people with PD. The device provides support to the ankle joint via a carbon fiber frame and mechanical drive cables, allowing for 30 degrees of ankle dorsiflexion and plantarflexion. The weighted design at the waist ensures the overall weight is only 3.3 kg. Its intelligent sensing system includes high-precision motion sensors (sampling data 100 times per second) and mechanical sensing elements, which continuously monitor the leg’s movement angle (with an error of less than 0.5 degrees), walking rhythm, and the interaction forces between the device and the body. Through a data fusion algorithm, the system accurately identifies the key phases in the walking cycle that require assistance (with an accuracy of 92%).

**Figure 1.**
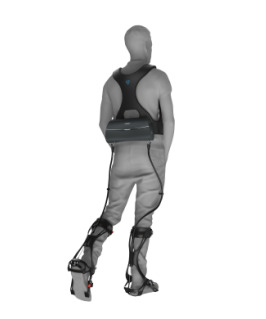
Yrobot Relink soft robotic exoskeleton used for soft robotic exoskeleton–assisted overground walking training.

Using an intelligent learning algorithm, the system automatically analyzes muscle electrical signals and changes in plantar pressure during walking, generating support forces that align with natural human movement during the later stages of gait (from heel-off to toe-off). The therapist can adjust the level of assistance in real time via a tablet and monitor gait symmetry indicators. The device is specifically designed with an anti-foot-drop safety mechanism, using a spring mechanism to restrict abnormal foot inversion, ensuring movement safety during use (Figure 1). Participants underwent a standardized gait training protocol, which was conducted in 3 phases: initial assessment—each participant’s baseline gait parameters (speed, stride length, and symmetry) were measured using a 3D motion capture system; training sessions—during each session, the level of assistance was dynamically adjusted based on real-time feedback from the system’s sensors, with the therapist modifying the support intensity according to the patient’s performance; and postassessment—after the training sessions, participants’ gait performance was reassessed, and changes in gait parameters, such as improvement in walking speed, step length, and symmetry, were recorded.

### Training Protocol

**Figure 2.**
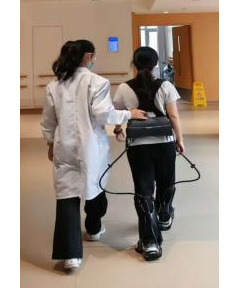
Photo of the soft robotic exoskeleton–assisted overground walking training procedure used in the study, which involves participants wearing the Yrobot Relink and engaging in controlled walking exercises designed to enhance gait stability and motor function in patients with Parkinson disease. The study was conducted over a 4-wk period at Shanghai Yangzhi Rehabilitation Hospital, with a total of 56 participants.

Both the CR group and the SRE group underwent a 4-week walking training program, with 5 sessions per week, each lasting 20 minutes, totaling 20 sessions. The assistive force of the exoskeleton (in Newtons) was set according to the participant’s body weight (in kilograms). Patients underwent evaluation and walking training 1 to 2 hours after medication intake to ensure they were in the “on” phase. The CR group received physical therapy and occupational therapy at the inpatient rehabilitation center, which included standard training in upper and lower limb strength, transfers, balance, walking, and activities of daily living. All physical therapy sessions were conducted by 2 rehabilitation physical therapists who had been trained in the intervention protocol prior to the study. During each training session, the physical therapists walked on either side of the participant to ensure safety (Figure 2).

### Outcome Measures

The researchers assessed the relevant indicators of both groups of participants at 3 time points: at baseline (before training), after 10 training sessions, and after 20 training sessions. The evaluations were performed by a physical therapist who was blinded to the specific group assignments and did not participate in any aspect of the training intervention.

- Motor function evaluation: Unified Parkinson Disease Rating Scale Part III (UPDRS-III) and its balance assessment.
- Nonmotor function evaluation: Montreal Cognitive Assessment and Modified Barthel Index.
- Balance ability evaluation: The limits of stability test was used to assess the participant’s ability to shift their center of gravity along the furthest possible boundary on the Balance Master system (NeuroCom International). Before the test, participants were instructed to extend their hips and knees to maintain an upright posture, with their feet stationary and hands placed vertically at their sides. During the test, participants were asked to lean their body in the directions of the computer screen (front, back, left, and right) for 10 seconds each, maintaining balance without falling. Limits of stability indicators include reaction time (RT), mean angular velocity, maximum center of pressure displacement, and directional control (DCL). The maximum center of pressure displacement is defined as the farthest distance the participant’s center of pressure moves during the tilt and is measured as the distance required to complete the task. Scores range from 0% to 100%, with higher percentages indicating better balance. DCL is assessed by measuring the amount of movement toward the target, compared to movements away from the target (external movement), and is defined as the ratio of the difference between expected movement and external movement to the expected movement. An integrated score across 8 directions was used for analysis. Scores range from 0% to 100%, with higher percentages indicating better motor control. The Sensory Organization Test includes a series of test conditions that assess stability and instability in both visual environments (open and closed eyes) and different states of the balance platform (fixed or mobile). These conditions are categorized as follows: condition 1 (eyes open, platform stable), condition 2 (eyes closed, platform stable), condition 3 (eyes open, platform stable, visual scene moves with center of gravity), condition 4 (eyes open, platform moves with center of gravity), condition 5 (eyes closed, platform moves with center of gravity), and condition 6 (eyes open, both platform and visual scene move with center of gravity) [5]. During the test, participants complete 6 progressive tasks under the guidance of the system. Each task specifically simulates the dominance of a single sensory system (eg, vision, proprioception, or vestibular sense) to quantify its role in dynamic balance control and compensatory effectiveness. The system automatically calculates the balance score based on the peak of the actual sway angle and theoretical sway angle, with higher scores reflecting better balance ability.

- Gait spatiotemporal parameters and kinematic data: The Yrobot Relink flexible exoskeleton robot collects spatiotemporal parameters (including step length, step speed, and swing phase proportion) and ankle joint angle indicators during participants’ self-paced walking.

### Statistical and Minimal Important Difference Analyses

Data analysis was performed using IBM SPSS Statistics (version 25.0; SPSS Inc). All continuous variables underwent normality tests. Data that followed a normal distribution were expressed as mean (SD), and intergroup comparisons were made using parametric tests. Nonnormally distributed data were presented as median (IQR), and categorical variables were expressed as counts (percentages), with intergroup comparisons made using nonparametric tests.

Within-group comparisons were conducted using paired sample *t* tests, while independent sample *t* tests were used for between-group comparisons. To explore the correlation between changes in ankle joint improvement after intervention and changes in primary clinical outcomes, correlation analyses were performed using Spearman rank correlation and Pearson chi-square tests. A *P* value of <.05 was considered statistically significant.

In addition to statistical analysis, the clinical significance of changes in primary clinical outcomes was evaluated by comparing these changes with the minimal clinically important difference (MCID). On the basis of previous research on gait interventions for patients with PD, the MCID threshold for walking speed change before and after intervention was defined as 0.05 m/s, which corresponds to a small meaningful change [28]. A significance level of α of .05 was used for all statistical analyses.

## Results

### Patient Characteristics

**Figure 3.**
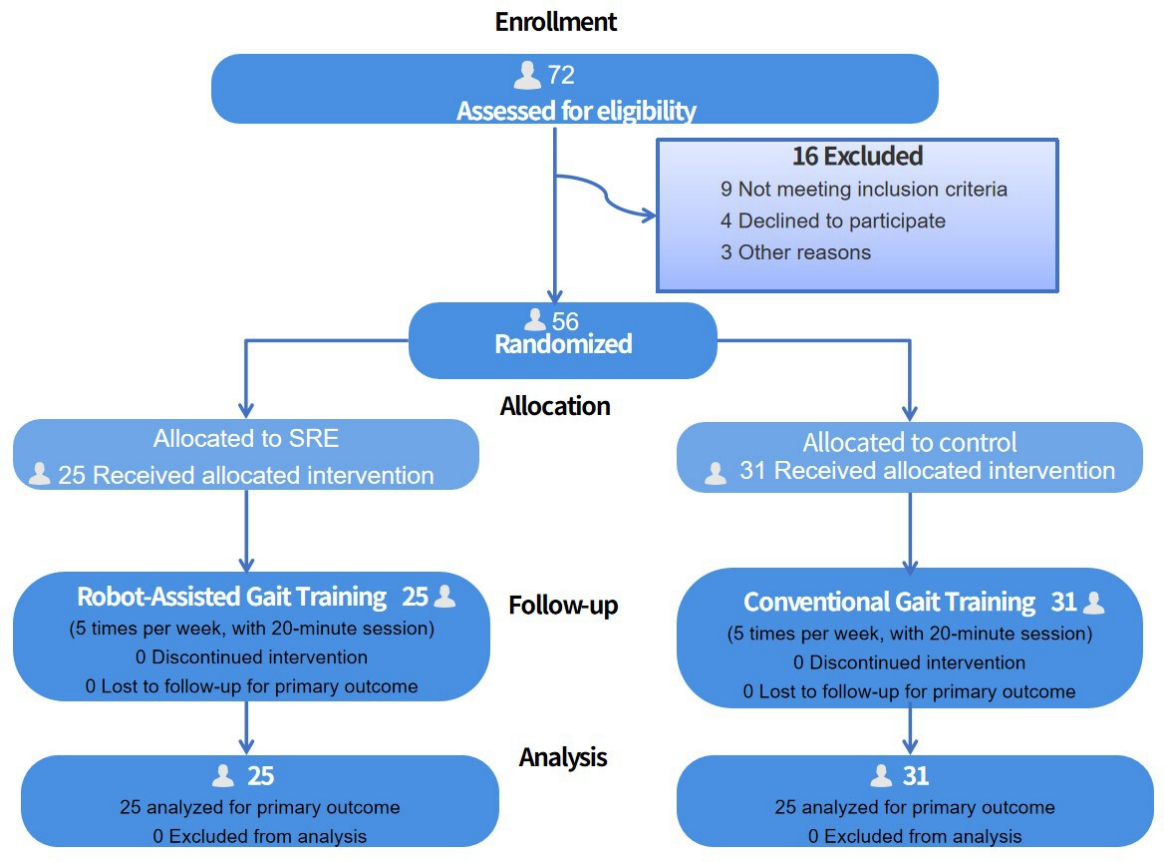
Research intervention method: Participants underwent a structured 4-wk program, with sessions held 5 times per week. SRE: soft robotic exoskeleton.

From July 2023 to May 2025, a total of 72 eligible patients were included in this study, with 16 (22.2%) patients excluded due to failure to meet the inclusion criteria and other reasons. Among the participants, 28 were men, and 28 were women. Statistical analysis of the baseline characteristics of both groups showed no significant differences (*P*>.05), indicating comparability between the groups, as presented in Table 1 and Figure 3. No serious adverse events occurred during the intervention for either group.

**Table 1.** General information of the participants.

Characteristics	SRE^a^ group (n=25)	CR^b^ group (n=31)	*P* value
Age (y), mean (SD)	66.76 (7.08)	70 (65,74)	.31
Height (cm), mean (SD)	167.20 (8.46)	166.26 (7.96)	.67
Weight (kg), mean (SD)	61.80 (6.68)	64 (53,65)	.59
H-Y^c^ (stage), mean (range)	3.0 (2.75-3.0)	3.0 (2.5-3.0)	.38
BMI (kg/m^2^), mean (SD)	22.11 (1.96)	21.89 (2.22)	.70
Comorbidities, n (%)			
Postoperative DBS^d^	5 (19.2)	6 (22.2)	.79
Diabetes mellitus	4 (15.4)	4 (14.8)	.85
Hypertension	6 (23.1)	9 (33.3)	.41
Cerebrovascular diseases	5 (19.2)	5 (18.5)	.95
Joint stiffness	12 (46.2)	14 (53.8)	.43
Levodopa-equivalent dose (mg/d), mean (SD)	332.7 (178.8)	369.0 (157.3)	.43
UPDRS^e^-III (score), mean (SD)	28.64 (11.20)	30.19 (11.97)	.62
UPDRS-III balance (score), mean (SD)	2.32 (1.28)	2.65 (1.11)	.31
Gait velocity (m/s)	Mean 0.55 (SD 0.27)	Median 0.52 (IQR 0.36-0.64)	.80
Left step length (m), mean (SD)	0.52 (0.28)	0.56 (0.24)	.59
Right step length (m), mean (SD)	0.54 (0.27)	0.56 (0.24)	.83
Left swing phase (%)	Median 27.50 (IQR 23.62-28.16)	Mean 25.36 (SD 4.75)	.35
Right swing phase (%), mean (SD)	26.43 (3.78)	26.07 (4.10)	.74
Left ankle plantarflexion (degree), mean (SD)	45.36 (7.65)	46.47 (7.88)	.87
Left ankle dorsiflexion (degree), mean (SD)	8.93 (3.36)	9.20 (2.54)	.82
Right ankle plantarflexion (degree), mean (SD)	46.00 (8.29)	47.87 (6.94)	.59
Right ankle dorsiflexion (degree), mean (SD)	8.79 (4.00)	10.00 (2.30)	.13
COP-Max^f^ (%), mean (SD)	51.96 (13.47)	51.55 (20.40)	.58
Reaction time (s), mean (SD)	1.35 (0.46)	1.47 (0.50)	.49
Angular velocity (degree per second), mean (SD)	1.94 (1.02)	2.20 (1.14)	.83
Directional control (%), mean (SD)	63.04 (13.47)	64.24 (15.73)	.83
SOT^g^ composite (score), mean (SD)	62.44 (10.85)	64.80 (11.28)	.79
BBS^h^ (score), mean (SD)	41.12 (6.05)	40.45 (9.36)	.75
Barthel Index (score), median (IQR)	77 (70-91)	85 (65-100)	.32
MoCA^i^ (score), mean (SD)	20.80 (5.20)	21.03 (5.14)	.87

a SRE: soft robotic exoskeleton device.

b CR: conventional rehabilitation.

c H-Y: Hoehn-Yahr.

d DBS: deep brain stimulation.

e UPDRS: Movement Disorder Society-Unified Parkinson disease rating scale.

f COP-Max: maximum center of pressure displacement.

g SOT: Sensory Organization Test.

h BBS: Berg Balance Scale.

i MoCA: Montreal Cognitive Assessment

All continuous variables underwent normality tests. Data following a normal distribution were expressed as mean (SD), while nonnormally distributed data were presented as median (IQR).

### Gait Spatiotemporal Parameters and Motor Function Outcome Measures

**Figure 4.**
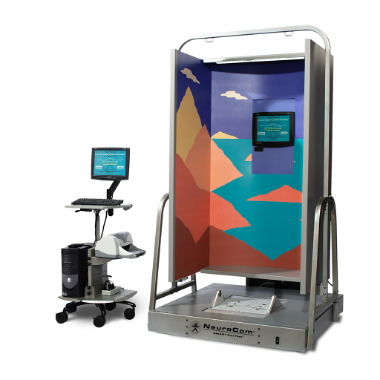
The Balance Master system used for assessing balance and motor function in participants before and after the intervention. This system measures postural stability, dynamic balance, and functional performance and was used as part of the outcome measures for both the soft robotic exoskeleton and conventional rehabilitation groups throughout the study.

To explore the mechanisms by which intervention measures affect gait and motor function, this study collected and analyzed spatiotemporal and kinematic parameters at 2 time points (preintervention and postintervention) during walking (Figure 4). Paired *t* tests revealed statistically significant differences between the 2 groups in motor assessment indicators (total UPDRS-III score: SRE, 95% CI −7.33 to −3.45; *P*<.001; and CR, 95% CI −6.01 to −4.07; *P*<.001) and its balance subscore (SRE: 95% CI −1.05 to −0.49; *P*<.001; and CR: 95% CI −0.81 to −0.39; *P*<.001), gait parameters (walking speed: SRE, 95% CI 0.17‐0.26; *P*<.001; and CR, 95% CI 0.11‐0.20; *P*<.001), step length (*P*<.001), left swing phase percentage (SRE: *P*=.01; and CR: *P*=.04), and right ankle plantar flexion angle (SRE: *P*<.001; and CR: *P*=.02) before and after intervention. However, significant improvements postintervention were observed only in the SRE group, with right swing phase percentage (95% CI 0.62‐2.62; *P*=.002), left ankle plantar flexion angle (*P*=.002), left ankle dorsiflexion angle (*P*<.001), and right ankle dorsiflexion angle (*P*=.01), indicating a specific advantage of the SRE intervention on these parameters, as detailed in Table 2.

**Table 2.** Within- and between-group comparisons of gait spatiotemporal parameters and motor function outcome measures in the soft robotic exoskeleton device (SRE) and conventional rehabilitation (CR) groups.^a^

Measure	SRE group (n=25)	CR group (n=31)	Within-group *P* value	Between-group *P* value
UPDRS^b^-III (score)				
Pre	30.19 (11.97)	28.64 (11.20)	—^c^	—
Post	24.81 (12.95)^d^	23.60 (10.56)^d^	<.001	.91
Post-pre	2.80 (3.98)	3.84 (2.30)	—	—
UPDRS-III balance (score)				
Pre	2.65 (1.11)	2.32 (1.28)	—	—
Post	1.87 (1.02)^d^	1.72 (1.02)^d^	<.001	.49
Post-pre	0.40 (0.58)	0.44 (0.51)	—	—
Gait velocity (m/s) %＞MCID^e^ (0.05m/s)	96%	41%		
Pre	0.54 (0.24)	0.55 (0.27)	—	—
Post	0.75 (0.24)^d^	0.71 (0.27)^d^	<.001	.04
Post-pre	0.23 (0.10)^f^	0.15 (0.12)	—	—
Left step length (m)				
Pre	0.56 (0.24)	0.52 (0.28)	—	—
Post	0.71 (0.25)^d^	0.63 (0.30)^d^	<.001	.19
Post-pre	0.15 (0.16)	0.11 (0.11)	—	—
Right step length (m)				
Pre	0.56 (0.24)	0.54 (0.27)	—	—
Post	0.71 (0.24)^d^	0.66 (0.28)^d^	<.001	.47
Post-pre	0.15 (0.15)	0.12 (0.11)	—	—
Left swing phase (%)				
Pre	25.36 (4.75)	26.49 (4.01)	—	—
Post	26.92 (3.91)^g^	28.04 (4.48)^h^	.01; .04	.87
Post-pre	1.56 (3.05)	1.56 (3.47)	—	—
Right swing phase (%)				
Pre	26.07 (4.10)	26.43 (3.78)	—	—
Post	27.70 (3.52)^g^	27.83 (4.14)	.002; .06	.57
Post-pre	1.62 (2.72)	1.40 (3.50)	—	—
Left ankle plantarflexion (degree)				
Pre	46.47 (7.88)	45.36 (7.65)	.002; .11	.23
Post	49.27 (6.39)^g^	46.28 (8.66)	—	—
Post-pre	2.3 (4.24)	1.22 (1.77)	—	—
Left ankle dorsiflexion (degree)				
Pre	9.20 (2.54)	8.93 (3.36)	<.001; .06	.17
Post	11.67 (3.13)^d^	10.36 (3.84)	—	—
Post-pre	2.84 (1.46)	1.38 (2.84)	—	—
Right ankle plantarflexion (degree)				
Pre	47.87 (6.94)	46.00 (8.29)	<.001; .02	.74
Post	49.20 (6.39)^d^	47.42 (8.06)^h^	—	—
Post-pre	1.79 (2.75)	1.42 (1.91)	—	—
Right ankle dorsiflexion (degree)				
Pre	10.00 (2.30)	8.79 (4.00)	.01; .07	.34
Post	11.67 (2.94)^g^	9.86 (3.53)	—	—
Post-pre	1.79 (2.22)	1.07 (1.90)	—	—

a Compared to the control group (post-pre: postintervention minus preintervention). To clarify the within-group *P* values, the first value represents the SRE group, and the second value corresponds to the CR group. When only one value is reported, it indicates that the *P* value is the same for both groups.

b UPDRS: Movement Disorder Society-Unified Parkinson disease rating scale.

c Not applicable.

d *P*<.001.

e MCID: minimal clinically important difference.

f *P*<.05.

g *P*<.01.

h Compared to pretreatment (pre), *P*<.05.

Independent sample *t* tests showed that the improvement in walking speed (Δ0.23 [0.10 m/s] vs Δ0.15 [0.12 m/s]) in the SRE group was significantly superior to the control group (*P*=.04), suggesting that SRE effectively restores motor function in people with PD, as presented in Table 2.

**Figure 5.**
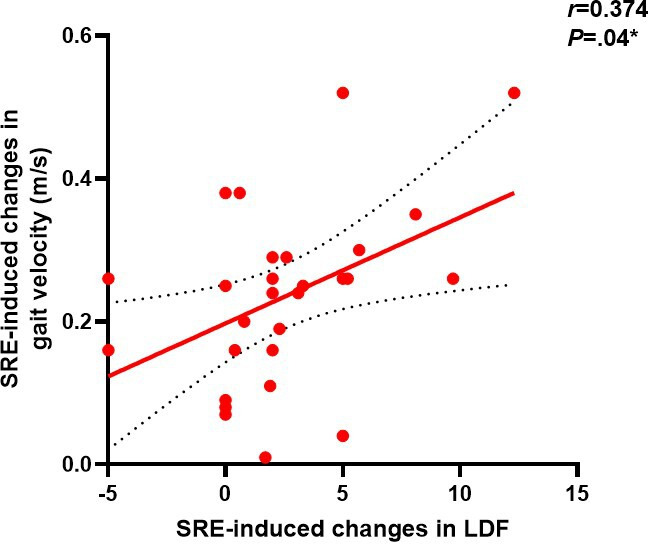
Correlations in soft robotic exoskeleton (SRE)–induced changes in gait spatiotemporal parameters and correlation of SRE-induced changes (post-pre) in left ankle dorsiflexion (LDF; x-axis) and SRE-induced changes in gait velocity (y-axis). *P<.05.

**Figure 6.**
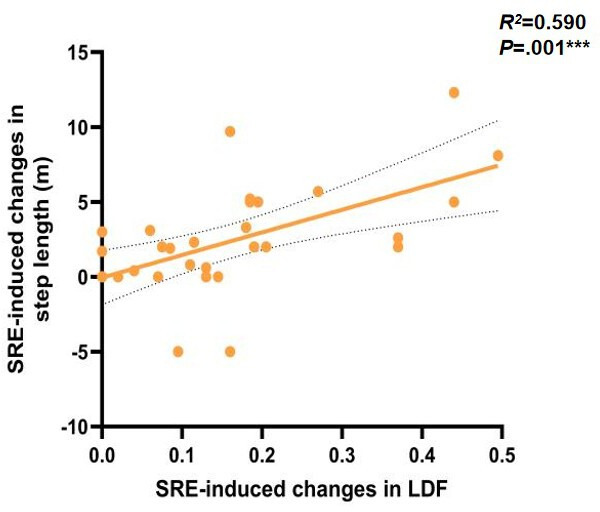
Correlations in soft robotic exoskeleton (SRE)–induced changes in gait spatiotemporal parameters and correlation of SRE-induced changes in left ankle dorsiflexion (LDF; x-axis) and SRE-induced changes in step length (y-axis). ***P<.001.

**Figure 7.**
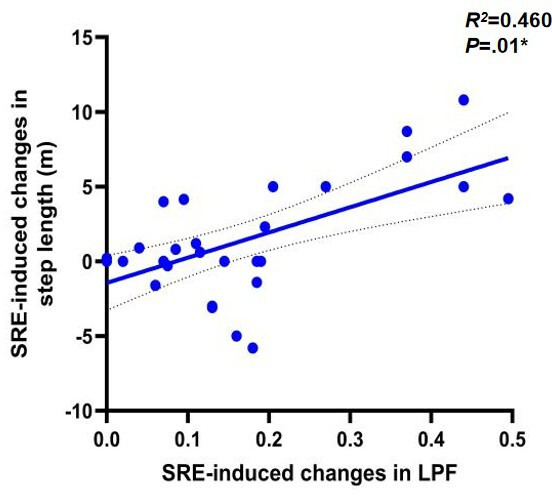
Correlations in soft robotic exoskeleton (SRE)–induced changes in gait spatiotemporal parameters and correlation of SRE-induced changes in left ankle dorsiflexion (LPF; x-axis) and SRE-induced changes in step length (y-axis). *P<.05.

**Figure 8.**
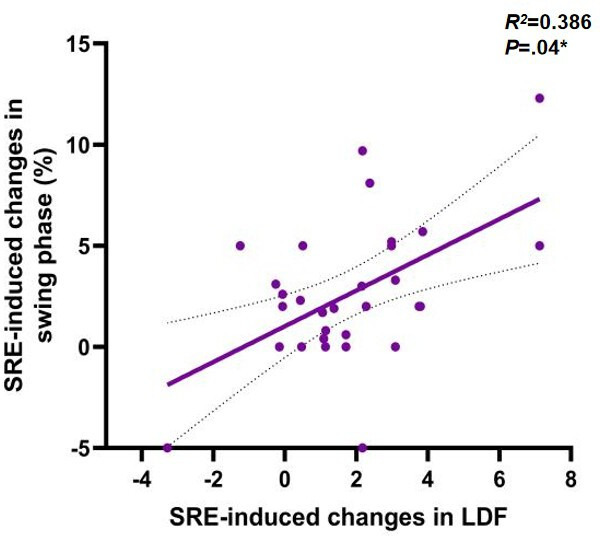
Correlations in soft robotic exoskeleton (SRE)–induced changes in gait spatiotemporal parameters and correlation of SRE-induced changes in LDF (X) and SRE-induced changes in swing phase (Y). *P<.05.

**Figure 9.**
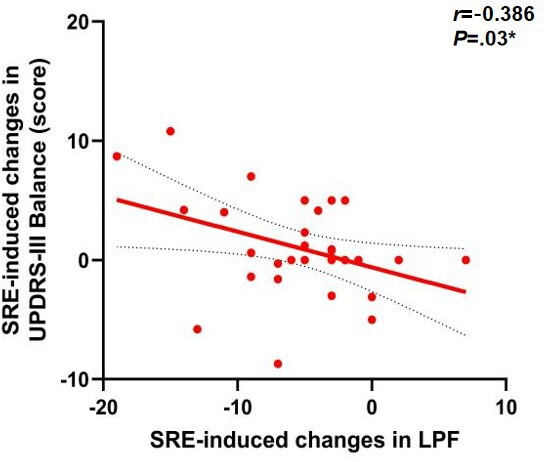
Correlations in soft robotic exoskeleton (SRE)–induced changes in Unified Parkinson Disease Rating Scale Part III (UPDRS-III) balance score. *P<.05.

**Figure 10.**
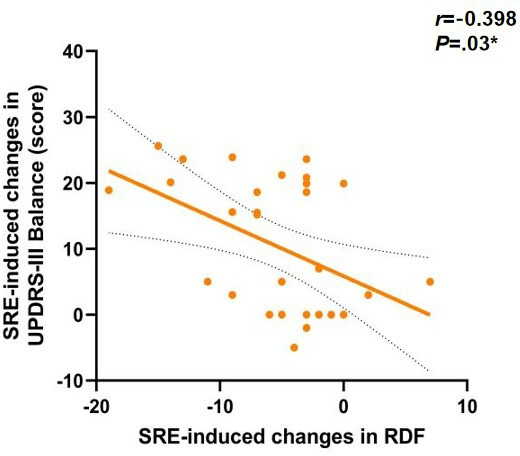
Correlations in soft robotic exoskeleton (SRE)–induced changes in Unified Parkinson Disease Rating Scale Part III (UPDRS-III) balance score. RDF: right ankle dorsiflexion. *P<.05.

To further investigate the potential mechanisms underlying gait changes induced by SRE, linear regression models were used to assess the correlation between improvements in ankle joint function and changes in outcome measures. The results first showed a significant positive correlation between the degree of left ankle dorsiflexion improvement and the increase in walking speed (*r*=0.374, *P*=.04), as shown in Figure 5. Left ankle dorsiflexion (ρ=0.59; *P*=.001), as shown in Figure 6 and plantar flexion (ρ=0.46; *P*=.01), as shown in Figure 7, improvements were both significantly correlated with increased step length, and left ankle dorsiflexion improvement was significantly correlated with an increase in swing phase percentage (ρ=0.386; *P*=.04), as shown in Figure 8. No significant correlations were found between other variables. It is noteworthy that post-SRE intervention, improvement in right ankle function was not significantly correlated with any of the measured indicators. Furthermore, the results revealed significant negative correlations between improvements in left ankle plantar flexion (*r*=−0.386; *P*=.03), as shown in Figure 9, and right ankle dorsiflexion (*r*=−0.398; *P*=.03), as shown in Figure 10, and the decrease in UPDRS-III balance scores.

### Balance Evaluation and Nonmotor Symptom Outcome Measures

To investigate the mechanisms by which intervention measures impact balance and nonmotor symptoms, this study collected and analyzed balance parameters and clinical scales at 2 time points (preintervention and postintervention). Paired *t* tests revealed statistically significant differences between the 2 groups in balance assessment indicators (average walking speed: SRE, *P*<.001; and CR, *P*=.02; DCL: SRE, *P*<.001; and CR, *P*=.01), sensory integration total score (*P*<.001), BBS scale (*P*<.001), quality of life (*P*<.001), and cognitive ability indicators (*P*<.001) before and after the intervention. However, significant improvements postintervention were observed only in the SRE group, with maximum movement distance (*P*<.001) and RT (*P*=.02), suggesting that SRE intervention has a specific advantage for these parameters, as detailed in Table 3.

**Table 3.** Within- and between-group comparisons of balance evaluation and nonmotor symptom outcome measures in the soft robotic exoskeleton (SRE) and conventional rehabilitation (CR) groups.^a^

Measure	SRE group (n=25)	CR group (n=31)
COP^b^-Max (%)		
Pre	51.55 (20.40)	51.96 (13.47)
Post	58.18 (18.99)^c^	55.26 (13.81)
Post-pre	7.70 (6.92)^d^	3.65 (5.76)
Reaction time (s)		
Pre	1.47 (0.50)	1.35 (0.46)
Post	1.22 (0.46)^e^	1.27 (0.46)
Post-pre	0.34 (0.27)^d^	0.06 (0.42)
Mean angular velocity (deg/sec)		
Pre	2.20 (1.14)	1.94 (1.02)
Post	2.55 (1.03)^f^	2.40 (1.36)^e^
Post-pre	2.54 (1.05)	2.30 (1.18)
Directional control (%)		
Pre	64.24 (15.73)	63.04 (13.47)
Post	72.19 (15.29)^c^	69.00 (9.90)^f^
Post-pre	7.95 (7.30)	6.76 (10.09)
SOT^g^ Composite (score)		
Pre	64.80 (11.28)	62.44 (10.85)
Post	70.86 (10.15)^d^	66.92 (10.65)^d^
Post-pre	6.33 (2.76)	4.76 (4.01)
BBS^h^ (score)		
Pre	40.45 (9.36)	41.12 (6.05)
Post	43.58 (9.27)^c^	45.44 (4.85)^c^
Post-pre	3.13 (3.00)	4.32 (2.90)
Barthel Index (score)		
Pre	81.13 (17.15)	76.68 (15.91)
Post	87.97 (11.67)^c^	83.96 (13.02)^c^
Post-pre	6.84 (7.14)	7.28 (5.11)
MoCA^i^ (score)		
Pre	21.03 (5.14)	20.80 (5.20)
Post	22.26 (4.77)^c^	22.00 (4.57)^c^
Post-pre	1.23 (1.23)	1.20 (1.08)

a Compared to the control group (post-pre: postintervention minus preintervention).

b COP-Max: maximum center of pressure displacement.

c *P*<.001.

d *P*<.05.

e Compared to pretreatment (pre), *P*<.05.

f *P*<.01.

g SOT: Sensory Organization Test.

h BBS: Berg Balance Scale.

i MoCA: Montreal Cognitive Assessment.

Independent sample *t* tests indicated that the improvement in maximum movement distance (Δ7.70, 6.92% vs Δ3.65, 5.76%) was significantly greater in the SRE group compared to the control group (*P*=.048), and the improvement in RT (Δ0.34, 0.27 s vs Δ0.06, 0.42 s) was also significantly superior in the SRE group (*P*=.04). This improvement may be related to the unique neuromuscular activation patterns or central-peripheral coordination mechanisms specific to the SRE intervention, as presented in Table 3.

## Discussion

### Principal Findings

This RCT aimed to investigate the clinical efficacy of an ankle-targeted SRE system in improving gait abnormalities and balance impairments in people with PD. To the best of our knowledge, no previous study has specifically assessed the value of SRE-assisted gait training for functional rehabilitation in PD [20], although there have been studies on robot-assisted rehabilitation for gait and balance in people with PD [20]. After a 4-week, 20-session standardized intervention, the SRE group showed significant intragroup improvements in both motor and nonmotor function indicators, confirming the potential of robot-assisted rehabilitation to address multidimensional functional impairments in PD. The main benefits of SRE-assisted walking training appear to lie in optimizing gait dynamics and enhancing balance function.

This study achieved important findings in gait parameter optimization, consistent with previous research highlighting the role of robotic interventions in gait rehabilitation for people with PD [24]. After 4 weeks of clinical training, both groups showed improvements in gait function, but the SRE group demonstrated a significantly greater increase in walking speed, with a higher MCID achievement rate compared to the control group. This result aligns with the conclusions of a meta-analysis of 7 RCTs on robot-assisted gait training, which found that robot-assisted gait training in any form significantly improves walking speed [29]. Notably, postintervention, the SRE group exhibited a greater increase in ankle dorsiflexion-plantarflexion range of motion and effectively corrected the compensatory prolonged support phase in people with PD. The proportion of the right lower limb swing phase increased in the SRE group, while no significant change was observed in the control group. These biomechanical improvements were closely related to enhanced ankle function. Correlation analysis revealed a positive association between increased ankle dorsiflexion and improvements in step length, walking speed, and swing phase duration.

The mechanisms underlying these results can be attributed to the dual regulatory effect of SRE intervention. At the peripheral biomechanical level, this is attributed to SRE’s ankle joint-driven assistive system. Research indicates that ankle dorsiflexor strength is a key determinant of walking speed [30], and in our findings, an increase in ankle dorsiflexion range was significantly positively correlated with walking speed. SRE effectively modulates the spatiotemporal coupling between the center of pressure trajectory and stride rhythm, reducing abnormal cocontraction of the ankle dorsiflexors and plantarflexors [31], thus surpassing traditional rehabilitation training in improving ankle joint mobility and supporting high-intensity repetitive gait training. Additionally, based on the central pattern generator hypothesis of walking control, SRE’s fixed gait rhythm input integrates proprioceptive feedback from the ankle Golgi tendon organ and hip muscle spindles [32,33], activating spinal central pattern generators to produce coordinated muscle activation patterns. This not only explains the 93.0% MCID achievement rate in walking speed but also strengthens the efficiency of the stance-to-swing phase transition, enhancing motor control automation.

The neuroplasticity mechanism supports the effectiveness of SRE, as high-intensity, repetitive tasks have been shown to induce brain reorganization in patients with PD [34]. The study further validated that early, task-oriented, high-intensity, and high-repetition training can significantly promote neuroplasticity changes [35], providing peripheral and central mechanistic support for the advantages of SRE intervention in early stages of PD.

In terms of postural control function, the SRE group demonstrated greater improvements compared to the control group. In the stability limit test, rotational response time decreased more substantially in the SRE group than in the CR group. The potential mechanism behind this improvement may be related to the activation of ankle strategies by the SRE intervention [36], achieved through high-precision proprioceptive feedback [37]. This enhances the rapid response of the ankle joint’s surrounding muscles (eg, the tibialis anterior), reversing delayed ankle dorsiflexion in people with PD and reducing postural preparation time, thereby optimizing the gait-posture coordination pattern [36]. Additionally, due to muscle rigidity, reduced joint mobility, and decreased axial flexibility, people with PD often exhibit poor rotational kinematic parameters [13]. Following SRE intervention, overall rotational speed and direction control showed greater improvement in the SRE group. Notably, the SRE group demonstrated a more pronounced increase in maximum rotational distance compared to the CR group, indicating enhanced dynamic stability. This metric reflects the maximal ability of people with PD to shift their center of mass without falling, and its significant improvement suggests enhanced dynamic stability. Under normal physiological conditions, individuals adopt an ankle strategy in response to small perturbations and a hip strategy for larger disturbances [38]. In the pathological state of PD, however, due to basal ganglia-thalamus-cortex loop dysfunction [38], the ability to apply the ankle strategy is significantly diminished, forcing premature use of the less efficient hip strategy [39], leading to trunk rigidity and limited center-of-mass adjustment [38,39]. The significant improvement in the “maximum rotational distance” in the SRE group may indicate that SRE enhanced the ankle joint’s resistance to perturbation, extended the boundaries of dynamic stability, improved the contraction efficiency of ankle muscles, delayed the threshold for initiating the hip strategy, and possibly optimized sensorimotor integration by improving proprioceptive input accuracy [5]. These results suggest that SRE may enhance postural stability through ankle joint support and improve PD-specific balance control dysfunction, potentially enhancing overall balance performance in people with PD. Further research is needed to confirm these effects in larger, more diverse populations. Additionally, the postural control dysfunction in people with PD may be linked to a fear of falling [40]. SRE might reduce the fear of falling in people with PD. Some scholars have suggested that robot-assisted walking may generate external rhythmic cues through proprioceptive feedback in a fixed mode, compensating for the internal rhythm deficits caused by basal ganglia circuit abnormalities in people with PD, thereby aiding postural control [41]. This “bottom-up” intervention strategy provides a novel target for breaking the PD posture-gait-activity vicious cycle.

It is noteworthy that the SRE group also showed improvements in cognitive symptoms and quality of life. The active participation in training has always been a critical factor in neurorehabilitation. Compared to platform-based robots, the gait training used in SRE allows patients to walk actively in a natural environment, capturing gait initiation intentions (such as heel strike angle and pressure center displacement) in real time through an inertial measurement unit, and providing assistive torque based on gait phases. This “intent-action closed-loop feedback” not only enhances neuromuscular engagement but also optimizes dynamic balance ability, thereby more effectively activating the motor cortex [42,43]. A study using electroencephalogram found that the frontal-central-parietal network is a potential neural marker for robot-assisted walking training to improve motor relearning and adaptation, promoting gait initiation and maintenance through activation of cortical and parietal motor pathways [44].

These findings have clear clinical implications. When providing rehabilitation interventions for people with PD, particularly those with balance disorders and fall risks, clinicians should pay close attention to the evaluation and management of ankle joint mobility limitations. This study suggests that the degree of improvement in ankle joint mobility can serve as a valuable predictive indicator for assessing and predicting the potential therapeutic effects on overall dynamic balance function (especially direction control) following SRE-assisted training or other ankle-focused interventions. Furthermore, to maximize rehabilitation benefits, future rehabilitation plans could incorporate more comprehensive approaches. These might combine technologies such as SRE, which provide precise joint assistance, with training methods focused on sensory integration, core stability, or gait coordination, in order to create synergistic effects that more comprehensively promote balance recovery and restoration in people with PD.

### Limitations

While this study provides promising evidence for the efficacy of SRE-assisted gait training in people with PD, several limitations must be acknowledged. First, the relatively small sample size may limit the statistical power and generalizability of the results. However, this prospective trial serves as a foundational step, offering an effect size estimate that could inform future large-sample RCTs designed to further validate and refine the intervention. Second, the absence of long-term follow-up data prevents an assessment of the durability of the SRE intervention effects, as well as the potential cumulative benefits of neuroplasticity. Long-term studies incorporating advanced imaging techniques, such as resting-state functional MRI, alongside serum neurotrophic factor analyses, could provide valuable insights into the dynamics of neural remodeling and the sustained impact of robotic rehabilitation. Finally, future research should delve deeper into the biomechanics of the SRE intervention. Integrating 3D gait analysis systems with surface electromyography would not only enhance our understanding of the mechanistic pathways involved but also refine therapeutic strategies to maximize the benefits of exoskeleton-assisted rehabilitation for people with PD. In a broader sense, these studies could contribute to the development of more personalized, evidence-based rehabilitation protocols, ultimately improving the quality of life for individuals living with PD.

### Conclusions

In conclusion, our findings confirm that integrating a soft exoskeleton robot (SER) into CR yields significant and clinically meaningful improvements in gait, balance, and broader functional outcomes for people with PD. The innovative use of a lightweight, adaptive SER—capable of real-time assistance during walking—fills a critical gap in evidence regarding modern wearable technologies for PD. Unlike earlier research that predominantly examined rigid exoskeletons or single-dimension effects, our study used a multifaceted evaluation (kinematic, kinetic, and clinical measures) and identified a mechanistic link: enhanced ankle dorsiflexion contributes to gains in stride length and gait speed. This advances the field by providing a replicable, evidence-based framework for incorporating SER into neurorehabilitation protocols. From a real-world perspective, the observed improvements in gait symmetry, balance control, and daily living activities suggest that SER can be feasibly implemented in clinical settings to help patients maintain mobility and autonomy, thereby addressing a core challenge in PD management. These results underscore the potential of soft robotics to transform rehabilitation practice and warrant further multicenter trials with longer follow-up.

## Supplementary material

10.2196/82629 Checklist 1 CONSORT checklist.

## Abbreviations

CONSORT: Consolidated Standards of Reporting Trials
CR: conventional rehabilitation
DCL: directional control
IRB: Institutional Review Board
MCID: minimal clinically important difference
PD: Parkinson disease
RCT: randomized controlled trial
RT: reaction time
SER: soft exoskeleton robot
SRE: soft robotic exoskeleton
UPDRS-III: Unified Parkinson Disease Rating Scale Part III
